# Floral nectary and osmophore of *Epipactis helleborine* (L.) Crantz (Orchidaceae)

**DOI:** 10.1007/s00709-018-1274-5

**Published:** 2018-06-09

**Authors:** Agnieszka K. Kowalkowska, Michalina Pawłowicz, Patrycja Guzanek, Agnieszka T. Krawczyńska

**Affiliations:** 1 Department of Plant Cytology and Embryology, University of Gdańsk, Wita Stwosza 59, 80-308 Gdańsk, Poland; 2 Faculty of Materials Science and Engineering, Warsaw University of Technology, Wołoska 141, 02-507 Warsaw, Poland

**Keywords:** *Epipactis helleborine ssp. helleborine*, *Orchidaceae*, Floral morphology, Micromorphology, Anatomy, Ultrastructure, Nectary, Osmophore

## Abstract

The analysis of flowers collected at different stages of anthesis provides strong evidence to conclude that the shell-shaped hypochile and the knobs of epichile form a nectary. The scent comes from the aromatic constituents of nectar and the epichile tissue and the apices of all tepals (osmophores). The comparison between pollinated and unpollinated flowers revealed that the anthesis of unpollinated flowers lasted up to the 16th day. The nectariferous secretory cells formed single-layered epidermis and several layers of underlying parenchyma built by small, isodiametric cells with thin walls and dense cytoplasm, relatively large nuclei, supplied by collateral vascular bundles. During the floral lifespan, the residues of secreted material were higher on the hypochile cells. The lipoid-carbohydrate material and lipid globules in the cell walls and in the cytoplasm were localised. The abundance of starch grains was observed at the beginning of anthesis and their gradual reduction during the flower lifespan. At the end of anthesis in unpollinated flowers, the lipoid-carbohydrate-phenolic materials have been demonstrated. The phenolic material was the same as in plastoglobuli. The features such as irregular plasmalemma, the secretory vesicles that fuse with it, fully developed dictyosomes, numerous profiles of ER indicate vesicle-mediated process of secretion. The substances could be transported by vesicles to the periplasmic space via granulocrine secretion and then to the external surface. Both micro-channels and slightly developed periplasmic space were visible in the hypochile epidermis. This is the first time for anatomical survey of secretory tissue in pollinated and unpollinated flowers of *E. helleborine.*

## Introduction

The plant nectaries diverse greatly in topography, anatomy, ultrastructure and secretion processes. Nectar, an aqueous solution with sugars, is regarded as a trait that appeared in plant–animal coevolution facilitated pollen dispersal and indirect defence against herbivores. The pollen dispersal is promoted by floral nectaries, where nectar is produced close to the reproductive organs, whereas the defence against herbivores by extra-floral nectaries, where nectar is exuded on the vegetative parts and is not involved in pollination (Nepi 2017). In orchid flowers, the nectaries can be formed as shallow, labellar nectaries, nectar spurs growing out from the lip base or the fused sepals (van der Pijl and Dodson 1969), labellar callus (Davies et al. 2005) or as cuniculus embedded in ovary (Dressler 1990).

The species from genus *Epipactis* offer the superficial nectar on labellum, which mostly attracts nectar feeders (van der Pijl and Dodson 1969; Nilsson 1978): Diptera and Hymenoptera in *Epipactis palustris* (L.) Crantz (Jakubska-Busse and Kadej 2011); Diptera, Hymenoptera and Coleoptera in *Epipactis helleborine* (L.) Crantz (Jakubska et al. 2005a). In anatomically examined flowers of *Epipactis atropurpurea* Raf. (Pais 1987), the nectary is placed in the concave basal part of labellum called gutter (= hypochile). The shallow nectary of *E. palustris* (Kowalkowska et al. 2015) is formed on the central broad isthmus of hypochile. The nectariferous cells are also found on lip knobs. The main attractants identified in nectar of *E. palustris* are nonanal (pelargonaldehyde), decanal, eicosanol and its derivatives. The scent composition of nectar contained strong aromatic compounds as eugenol and vanillin (Jakubska-Busse and Kadej 2011). Whereas, the chemical analysis of the nectar in *E. helleborine* revealed that the main components are eugenol, 2,6-dimethoxy-4-(2-propenyl)phenol (methoxyeugenol), ethanol and 4-hydroxy-3-methoxybenzaldehyde (vanillin) (Jakubska et al. 2005b). 3-{2-{3-{3-(benzyloxy)propyl}-3-indol, 7,8-didehydro-4,5-epoxy3,6-d-morphinan and their derivatives are potentially narcotic compounds, which might be responsible for the pollinators’ behaviour described as “drunken insects” or “sluggish pollinators”, not excluding the effect of ethanol on insects (e.g. Ehlers and Olesen 1997, after Jakubska et al. 2005a). In the forest species, such as *E. helleborine*, it is claimed that such compounds influenced that the insects, occasionally visiting the flowers, remained longer in flowers and caused the flower pollination. Many alcohols identified in the nectar, i.e. ethanol, 2,2-diethoxyethanol, methanol, 2-hydroxy-benzenmethanol, 4-hydroxybenzenmethanol and pentadecanol, heptadecanol, eicosanol as well as benzyl alcohol, could suggest the result of fermentation process (Jakubska et al. 2005a), but the presence of compounds with antimicrobial properties, e.g. furfural and syringol, could block the development of the yeast, as the fermentation process of sugars under natural condition occurs very rarely. The floral colour rather has a small meaning in insects’ attraction, whereas the floral odour is the main first attractant for insects (Jakubska 2003). The characteristic floral smell is probably caused by eicosanoic acid methyl ester, tetracosanoic acid methyl ester, pentadecenoic acid methyl ester, hexadecenoic acid methyl ester and vanillin (Jakubska et al. 2005b). The hypothetical plant-pollinator interaction is divided in *E. helleborine* into two steps: the first insect reaction on long-distance attractant—scent, i.e. vanillin, furfural, ethanol, eugenol and their derivatives; the second alimentary short-distance attractant—nectar with narcotic constituents, i.e. morphinian derivatives and indole derivatives, caused the disorientation of flights known as “sluggish” pollinators effect (Jakubska et al. 2005b).

*Epipactis helleborine* (broad-leaved helleborine) is an opportunistic species, one of the most common colonisers in temperate Europe, with broad ecological amplitudes and short life cycles (Rewicz et al. 2016 and literature therein, Rewicz et al. 2017). The plant mainly grows in forest (deciduous, coniferous), on their edges and in clearings in woodland, up to 2000 m a.s.l. (Delforge 2006), also in the anthropogenic habitats (roadsides, cemeteries, poplar plantations, gravel pits, quarries, railway embankments, mine tailings, spontaneously in town parks and gardens) (Świercz 2004, 2006; Kiedrzyński and Stefaniak 2011; Kolanowska 2013). Although the floral chemistry, pollinator diversity and population studies of *E. helleborine* are well examined, this is the first time for anatomical survey of secretory tissue in pollinated and unpollinated flowers of broad-leaved helleborine. The aims of the present work are (a) to verify the presence of nectary in flowers; (b) to examine in detail the secretory tissue; (c) to discuss the anatomical results of pollinated and unpollinated flowers with reference to attract pollinators. Such results allow us to better understand the pollination biology of *E. helleborine*.

## Materials and methods

The pollinated and unpollinated flowers were collected from *Epipactis helleborine* ssp. *helleborine* (Table 1). The flowers were shaded by pink, with large and distinct knobs of the epichile and the concave hypochile at the base. Tissue samples were collected (voucher numbers UGDA 2014-001; UGDA 2014-002) in July 2014 from two localities:

- The first locality: Gdańsk Jelitkowo GPS: 1. N 54°25.134′ E 18°36.624′, 2. N 54°25.117′ E 18°36.677′, 3. N 54°25.126′ E 18°36.629′, 4. N 54°24.809′ E 18°37.398′;
- The second locality: Sopot Kamienny Potok GPS: 1. N 54°28.032′ E 18°33.110′, 2. N 54°28.052′ E 18°33.276′; 3. N 54°27.930′ E 18°33.230′; 4. N 54°27.887′ E 18°33.029′.

**Table 1:** The collected flowers of *Epipactis helleborine*

The day of anthesis	Locality	Method	Figure
The unpollinated flowers			
Bud (8 mm in length)	2nd locality (Sopot Kamienny Potok)	Toluidine Blue O (TBO) (control)	2a, b
		PAS	2c
Bud (before opening)	2nd locality (Sopot Kamienny Potok)	PAS	2d
		Aniline Blue Black (ABB)	2e
		Transmission Electron Microscopy (TEM)	2f, h
		TBO	2g
1st day	1st locality (Gdańsk Jelitkowo)	Light microscopy (LM)	1a, b
		Scanning electron microscopy (SEM)	1e
		PAS	3a, b
1st day	2nd locality (Sopot Kamienny Potok)	PAS	3c, d
	1st locality (Gdańsk Jelitkowo)	Auramine O	3f
		Auramine O	3h
2nd day	2nd locality (Sopot Kamienny Potok)	TEM	4a–c
3rd day	2nd locality (Sopot Kamienny Potok)	SEM	1f
		Neutral Red	1c, d
5th day	1st locality (Gdańsk Jelitkowo)	PAS	3e
	2nd locality (Sopot Kamienny Potok)	Auramine O	3g
		TEM	5a–d, 7d
16th day	2nd locality (Sopot Kamienny Potok)	ABB	4d
		TEM	4e–h
The pollinated flowers			
1st day, empty pollinia	1st locality (Gdańsk Jelitkowo)	TEM	6c–f
1st day, with own pollinia	2nd locality (Sopot Kamienny Potok)	TEM	6g–i
1st day, empty pollinia	1st locality (Gdańsk Jelitkowo)	TEM	7e, f
3rd day, empty pollinia	2nd locality (Sopot Kamienny Potok)	FeCl_3_	6a
		SBB	6b
		TEM	7a–c
14th day, empty pollinia	2nd locality (Sopot Kamienny Potok)	TEM	7g, h

The first locality was open, sunny, exposed to wind, slightly shaded by trees, close to bicycle path, near the Baltic Sea, in the edge of the forest. The second locality was more shaded and humid, with the static ecological conditions, placed in the edge of the forest. In both localities, the populations were divided into smaller parts with 10–30 plants in each part. The flowers were collected at different days of anthesis: from the bud stage until the 16th day, which could be divided into ranges: the beginning of anthesis—from buds until the 2nd day, the fully opened flower—the 3rd day, the end of anthesis—the 5th day for the 1st locality and the 14–16th days for the 2nd locality. Noteworthy is the fact that the beginning of July in the year 2014 was outstanding with high temperatures during the day, which influenced on withering of flowers—the 5th day on the 1st locality was the last day of anthesis. As we did not observe significant differences on histochemical and ultrastructural level from two localities, we focused on comparison between pollinated and unpollinated flowers.

The fresh flowers were observed under a Nikon SMZ1500 stereomicroscope. For the purpose of localization of osmophores, the whole, fresh flowers were immersed in an aqueous solution of 0.01–0.001% (*w*/*v*) neutral red for 20 min. Hand-cut sections of fresh lips were prepared and treated with Sudan III and IKI solution in order to detect lipids and starch, respectively.

Plant material was fixed in 2,5% (*v*/*v*) glutaraldehyde (GA) in 0.05 M cacodylate buffer (pH = 7.0). Material for light microscopy (LM), following fixation, was rinsed with cacodylate buffer and then dehydrated in an acetone or ethanol series. Subsequently, material dehydrated in acetone was embedded in epoxy resin (Spurr 1969), whereas material dehydrated in ethanol was embedded in methylmethacrylate-based resin (Technovit 7100, Heraeus Kulzer GmbH). Sections were cut with glass knives (1–5 μm thick) and mounted on glass slides. For light microscopy, semi-thin control sections were stained with 0.05% (*w*/*v*) aqueous sodium tetraborate solution (Toluidine Blue O, TBO, C.I. 52,040) (Feder and O’Brien 1968; Ruzin 1999). Aniline Blue Black (ABB, C.I. 20,470) was used for the detection of water-insoluble proteins (Jensen 1962). The Periodic Acid-Schiff reaction (PAS) was used to identify the presence of water-insoluble polysaccharides (Jensen 1962) and a 0.3% (*w*/*v*) ethanolic solution of Sudan Black B (SBB, C.I. 26,150) for lipid localization (Bronner 1975). The preparations were examined and photographed by means of a Nikon Eclipse E 800 light microscope and a Nikon DS-5Mc camera using the Lucia Image software. Auramine O (C.I. 41,000; 0.01% (*w*/*v*) solution in 0.05 M buffer Tris/HCl, pH = 7.2) was used to detect the presence of cuticle (Heslop-Harrison 1977), especially unsaturated acidic waxes and cutin precursors (Gahan 1984), and the staining reaction was examined by means of a Nikon Eclipse E800 fluorescence microscope equipped with filter: B-2A (EX 450–490 nm, DM 505 nm, BA 520 nm). The 10% (*w*/*v*) aqueous solution of FeCl_3_ was used to test for catechol-type dihydroxyphenols (Gahan 1984). The sections were observed using the differential interference contrast (DIC) imaging.

Following dehydration in an ethanol series, the samples were prepared for scanning electron microscopy (SEM) and subjected to critical-point drying using liquid CO_2_, coated with gold and examined using a Zeiss EVO 40 SEM microscope (Electron and Confocal Microscope Laboratory, Faculty of Biology, Adam Mickiewicz University of Poznań) at an accelerating voltage 12–14 kV.

For transmission electron microscopy (TEM), floral material was fixed in 2.5% (*v*/*v*) glutaraldehyde (GA) in 0.05 M cacodylate buffer (pH 7.0). The material was then post-fixed overnight in 1% (*w*/*v*) OsO_4_ in cacodylate buffer in a refrigerator and finally rinsed in buffer. After 1 h in a 1% (*w*/*v*) aqueous solution of uranyl acetate, the material was dehydrated with acetone and embedded in Spurr’s resin. Ultrathin sections were cut on a Leica EM UC 7 ultramicrotome with a diamond knife and stained with uranyl acetate and lead citrate (Reynolds 1963). The sections were examined using a JEOL JEM 1200EX transmission electron microscope (Warsaw University of Technology) at an accelerating voltage 120 kV.

## Results

**Fig. 1:**
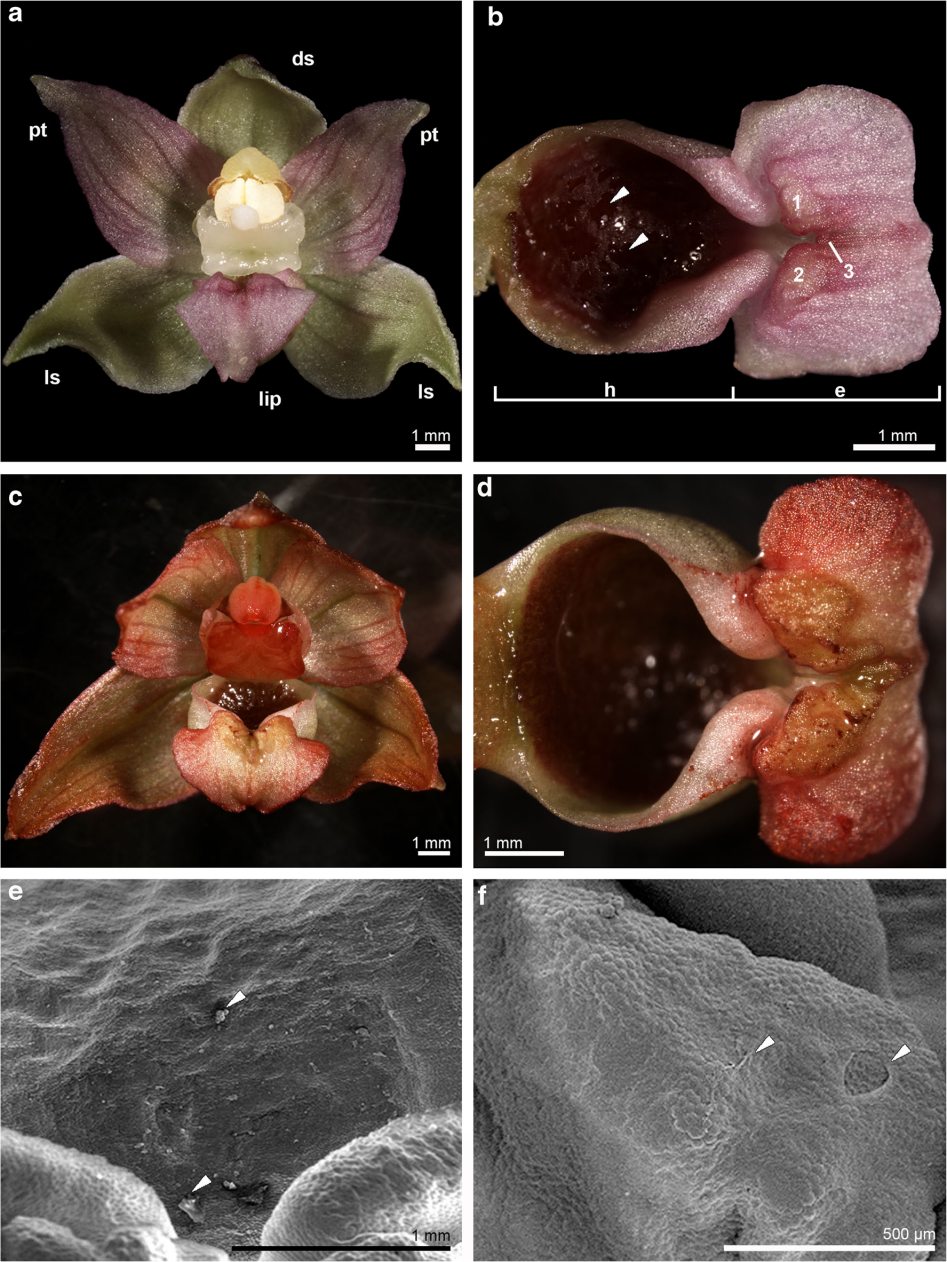
The unpollinated flowers of *Epipactis helleborine* (L.) Crantz. **a**, **b** The 1st day of anthesis. **a** Flower, *ds* dorsal sepal, *ls* lateral sepal, *pt* petal. **b** Lip divided on basal part (*h* hypochile) and apical part (*e* epichile), *1–3* knobs, secretion (*arrows*). **c**, **d** The 3rd day, neutral red test. **c** Flower with stained the apices of all tepals (sepals and petals), anther and stigma. **d** The positive reaction on lip margins of epichile and slightly knobs. **e**, **f** lip micromorphology (SEM). **e** The 1st day, the hypochile with undulated surface and few residues of secreted substances (*arrows*). **f** The 3rd day, the knob with a thin layer of secretory material (*arrows*)

The non-hinged lip was consisted from two parts (Fig. 1a, b): the cup-shaped concave basal part (the hypochile) and the heart-shaped apical part (the epichile) with the apex sharply bent down. A callus was placed at the base of epichile and composed of two lateral knobs and the third smaller central longitudinal knob.

### Unpollinated flowers

The osmophoric activity was detected on the apices of tepals (sepals and petals) and on the margins of epichile and slightly on the knobs, which stained positively in neutral red (Fig. 1c, d). The residues of secreted substances were observed on the hypochile and knobs surface, covered by a thin layer of secreted material (Fig. 1e, f). The epichile (with knobs) was built by smooth, polygonal cells. The surfaces from the 2nd locality were more pleated than in flowers from the 1st locality, which were caused by weather condition. In the first locality (sunny, open), the flowers usually bloomed until the 5th day and even if the tepals were dried, the lips were still fresh, especially the knobs. In the second shaded locality, we observed the unpollinated flowers even in the 16th day of anthesis.

**Fig. 2:**
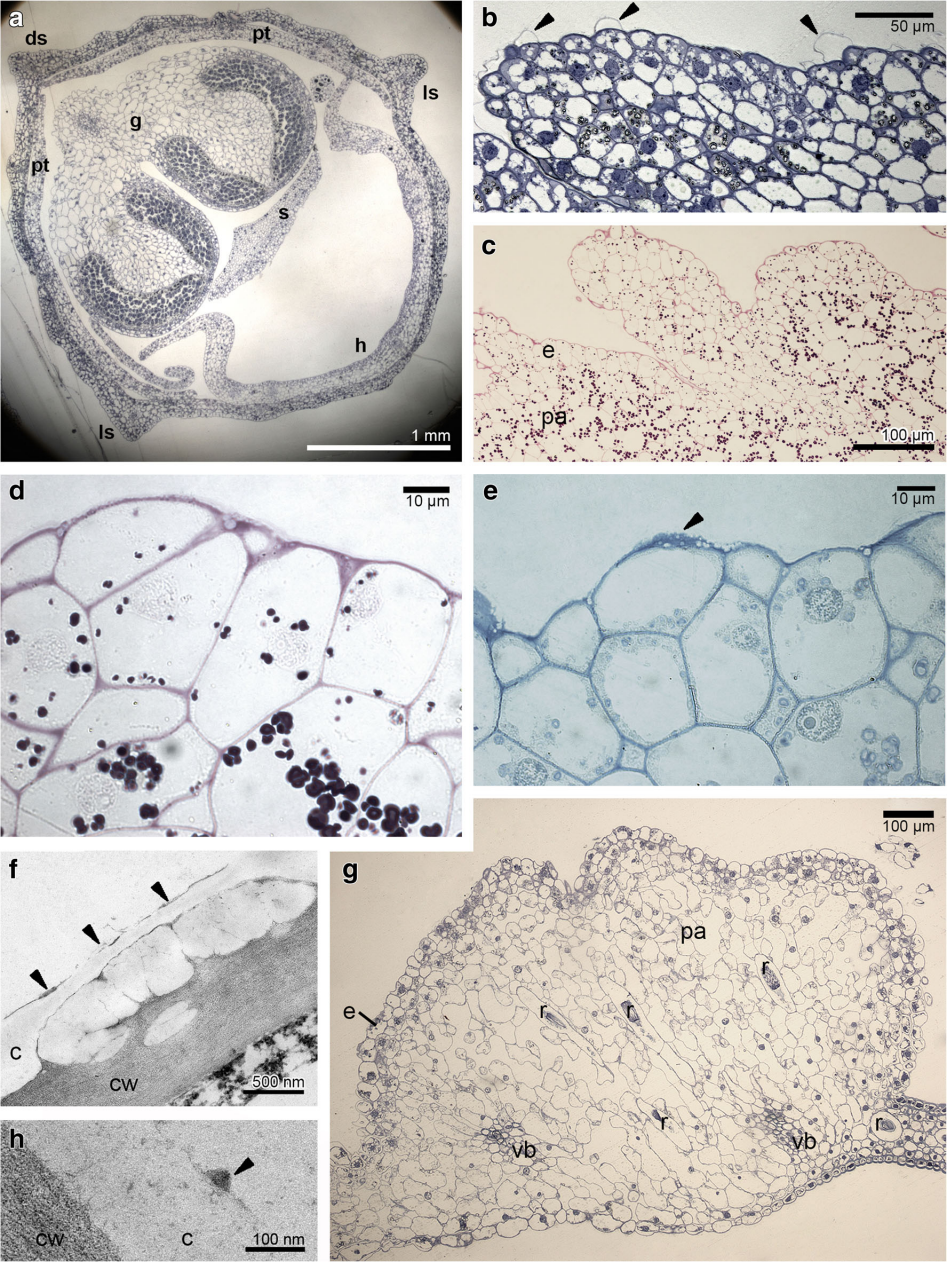
Histochemical tests of unpollinated flowers. **a**–**c** Bud (about 8 mm in length). **a** Cross-section (TBO). **b** The detail of hypochile with distended cuticle (*arrows*, TBO). **c** The hypochile: starch grains decreased in epidermis, numerous in parenchyma (PAS). **d**–**h** Bud—flower before opening. **d** Hypochile, the further stage of starch utilisation (PAS). **e** Proteins on the epidermal cells of hypochile (*arrow*, ABB). **f** The secretory material on the cuticle (*arrows*) and micro-channels of hypochile cells (TEM). **g** Knob: single-layered epidermis, 2–3 layers of subepidermis, parenchyma with intercellular spaces and collateral vascular bundles, idioblasts with raphides (TBO). **h** Few secretory material on the knob cells (TEM). *c* cuticle, *cw* cell wall, *ds* dorsal sepal, *e *epidermis, *g* gynostemium, *h* hypochile, *ls* lateral sepal, *pa *parenchyma, *pt *petal, *r* raphides in idioblast, *s* stigma, *vb* vascular bundle

**Fig. 3:**
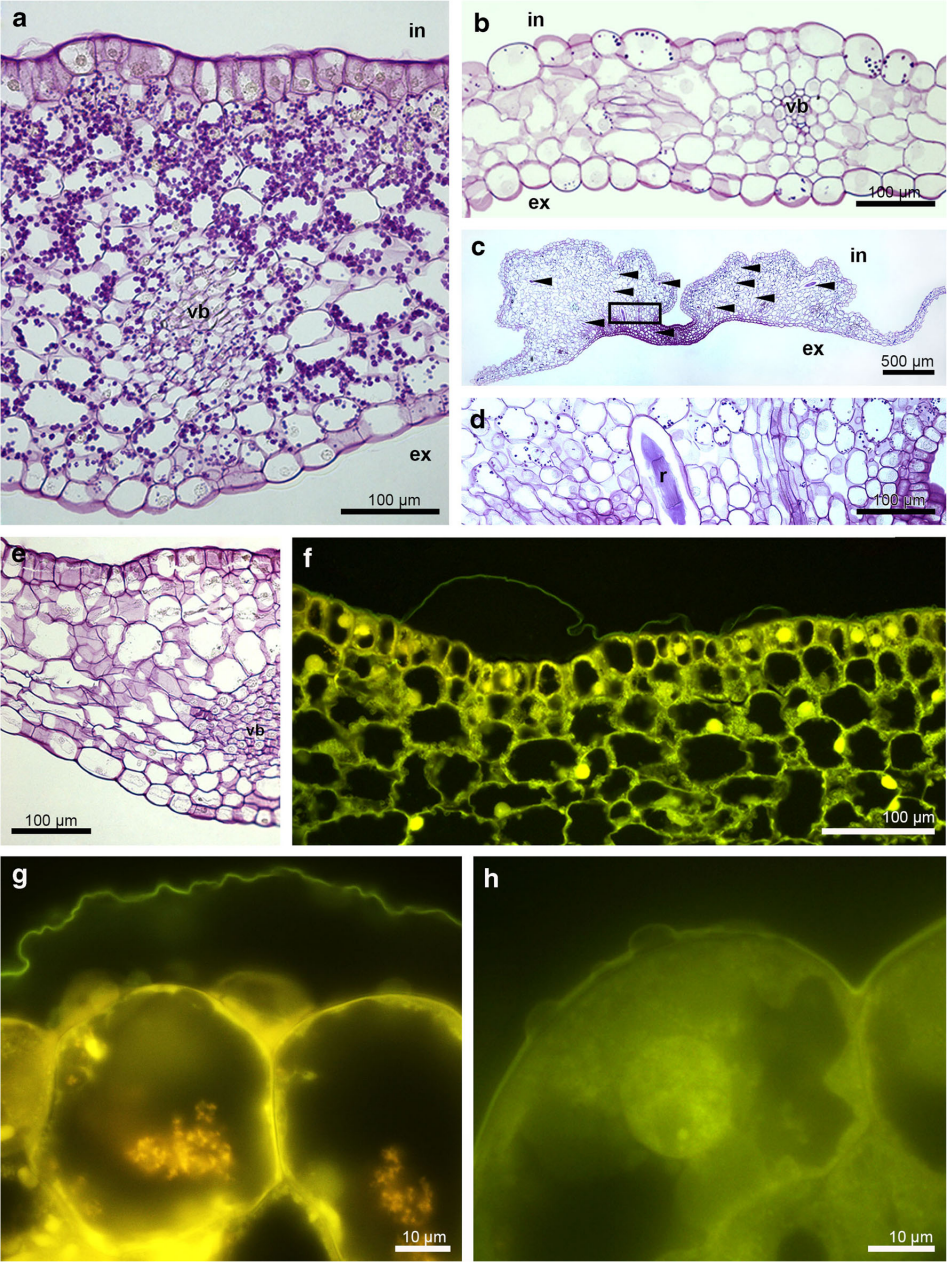
Histochemical tests. **a**–**e** Polysaccharides (PAS). **a** The 1st day of anthesis, the hypochile with starch grains. **b** The 1st day of anthesis, the epichile with few starch grains. **c** The 1st day of anthesis, the epichile with knobs, the idioblasts with raphides (*arrows*). **d** Detail of **c**, few starch grains, idioblast with raphides (*r*). **e** The 5th day, the hypochile without starch grains. **f**–**h** Auramine O. **f** The 1st day, the hypochile with distended cuticle. **g** The 5th day, the hypochile. **h** The 1st day, the knobs of epichile with small cuticle separation. *in* internal surface, *ex* external surface, *r* raphides, *vb* vascular bundle

**Fig. 4:**
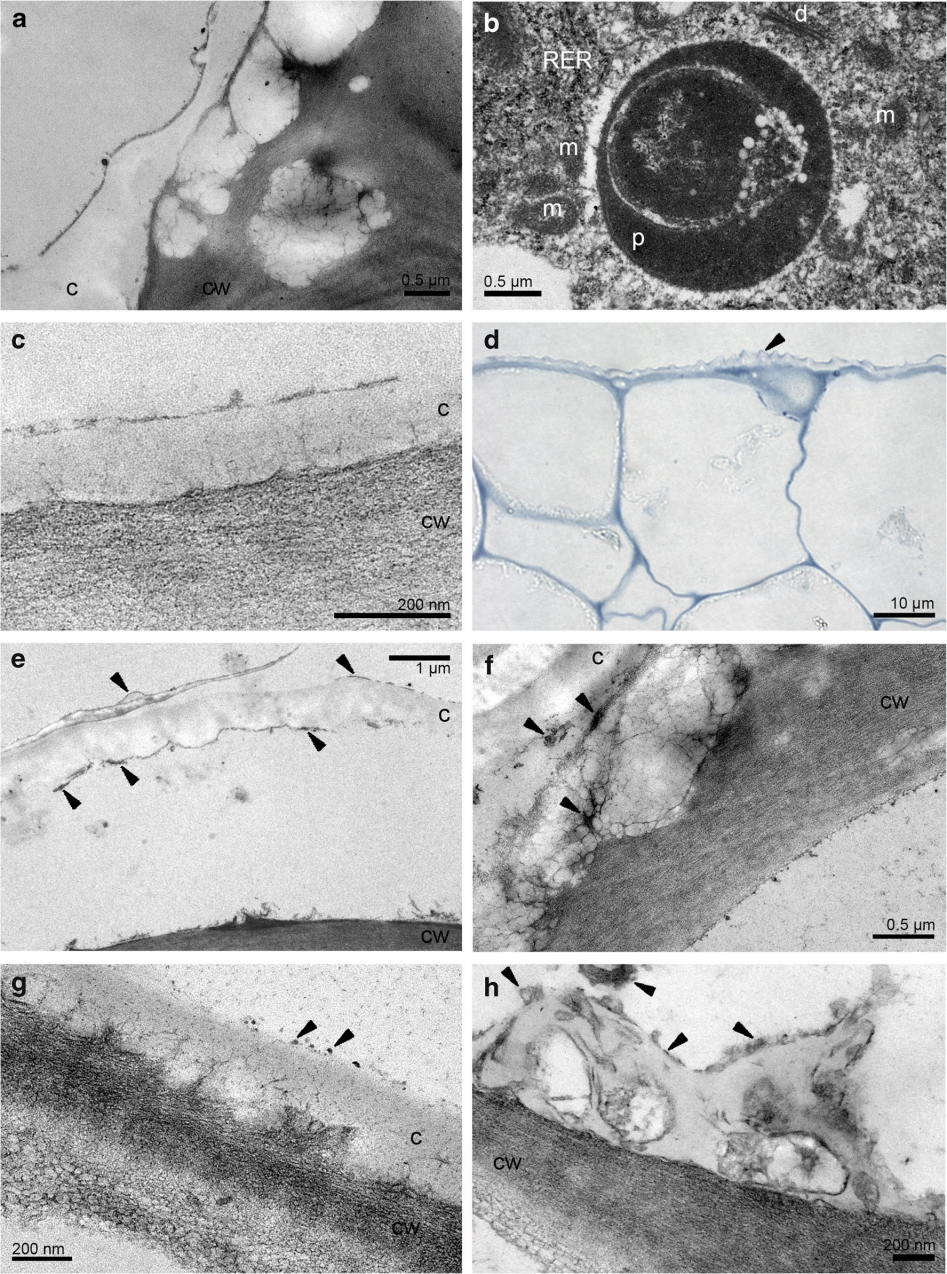
The unpollinated flowers. **a**–**c** Ultrastructure of flowers in the 2nd day of anthesis (TEM). **a** Secretory material on the cuticle and forming micro-channels of hypochile. **b** The dense cytoplasm with profiles of rough endoplasmic reticulum (RER), dictyosomes (*d*), mitochondria (*m*), plastid (*p*) of hypochile cells. **c** Few secretion on the knobs. **d**–**h** The 16th day of anthesis. **d** The proteins on the cuticle of hypochile cells (ABB). **e**, **f** Secretory material on the distended cuticle of hypochile with phenolic material (*arrows*) (TEM). **g**, **h** Secretion on the knobs with phenolic material (*arrows*) (TEM). *c *cuticle, *cw* cell wall

**Fig. 5:**
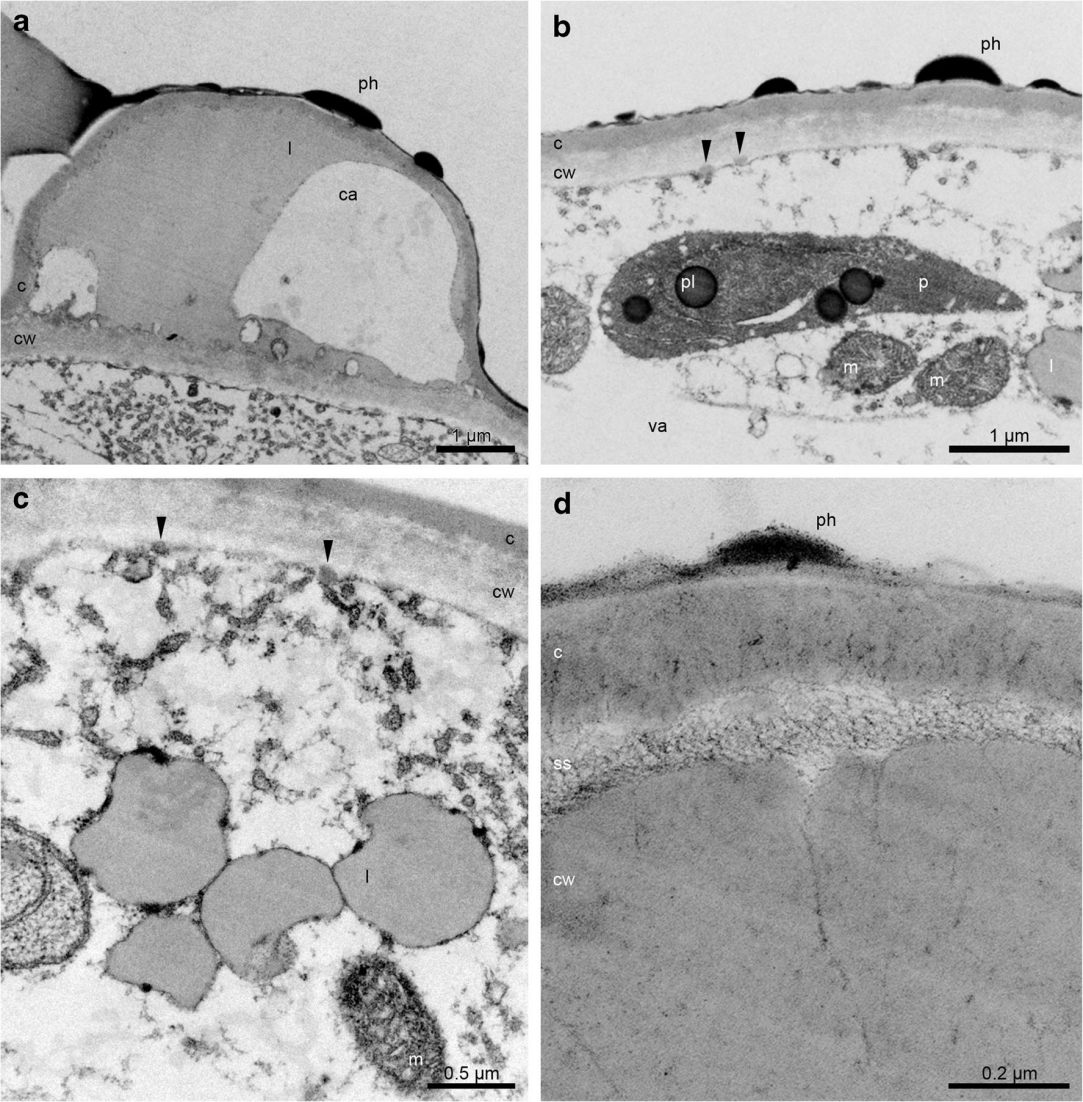
Ultrastructure of the knobs epidermal cells of epichile, the 5th day of anthesis. **a** The lipoid-carbohydrate bulges in subcuticular space. **a**, **b**, **d** Phenolic material on the cuticle surface. **b**, **c** Vesicles fusing with plasmalemma (arrows). **b** Phenolic material in plastoglobuli. **c** Lipid bodies in cytoplasm. **d** Few micro-channels in cuticle. *c* cuticle, *ca* carbohydrate material, *cw* cell wall, *l* lipid drop or lipoid material, *m* mitochondrion, *p* plastid, *ph* phenolic material, *pl* plastoglobuli, *ss* subcuticular space, *va* vacuole

The cross-sections of buds about 8 mm long revealed the pleated surface of hypochile with distended cuticle (Fig. 2a, b). The utilisation of starch grains started in bud stage from the epidermis as they were decreased comparing to the parenchyma (Fig. 2c) and other tepals (petals and sepals), which was also proved in the buds before opening (Fig. 2d). The ABB test revealed some proteins only on the epidermal cells of hypochile (Fig. 2e). The cuticle surface of hypochile cells was covered by secreted material, which had diffused through cuticle forming micro-channels (Fig. 2f). The tissue of knobs was built by a single layer of epidermis, 2–3 layers of subepidermis and parenchyma with intercellular spaces (Fig. 2g), with high number of idioblasts with raphides. However, the raphides were present in the all parts of flower. In this stage, the secretory material was found on the knobs in small amount (Fig. 2h). During the floral lifespan, the utilisation of starch was clearly visible—large number in parenchyma, but not found in the epidermis in the first day (Fig. 3a), few grains in epichile (Fig. 3b) and knobs (Fig. 3c, d), with the total utilisation in hypochile (Fig. 3e, the fifth day, the last day of anthesis in the first locality) and epichile. Also, the one to few lipid bodies (Sudan III), present in the beginning of anthesis, were absent in the fifth day. In the Auramine O test, the cuticle separation was also noticed (Fig. 3f, g; slightly on the knobs—Fig. 3h). The vascular bundles in lip tissue were collateral (Fig. 3a, b, e). The idioblasts with raphides were localised in the whole floral parts, however in greater number in the knobs (Fig. 3c, d). TEM research showed the residues of secretory materials on hypochile cells (Fig. 4a) and slightly on knobs (Fig. 4c). The plastids with internal membranes, mitochondria, dictyosomes, profiles of RER occurred in dense cytoplasm (Fig. 4b). In the 16th day of anthesis (2nd locality), the proteins were detected on the cuticle of hypochile cells (Fig. 4d). The secretory materials were also present under the cuticle and on the surface of hypochile and knobs, with phenolic content (Fig. 4e–h). Similarly, but in the fifth day (the last day of floral lifespan on the first locality), the lipoid-carbohydrate bulges in subcuticular space and phenolic material on the cuticle surface on knobs appeared (Fig. 5a, b, d), phenolic material in plastoglobuli (Fig. 5b) and lipid bodies in cytoplasm (Fig. 5c). The vesicles were fused with plasmalemma (Fig. 5b, c). Few micro-channels were noticed in cuticle (Fig. 5d).

### Pollinated flowers

**Fig. 6:**
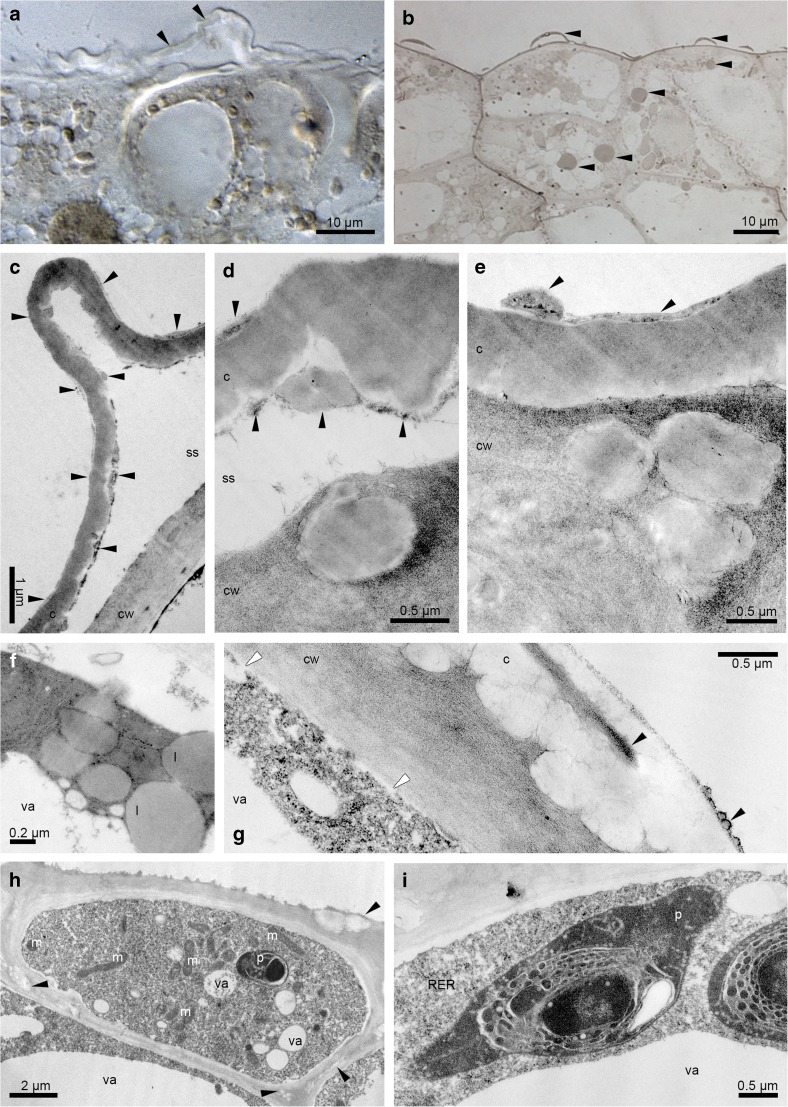
The pollinated flowers. **a**, **b** Histochemical tests, the 3rd day of anthesis. **a** Hypochile - the test for presence of dihydroxyphenols (FeCl_3_) with slight reaction of plastids (probably plastoglobuli) and thin layer on cuticle (*arrows*). **b** The knobs of epichile with lipid bodies and lipoid material on the cells (*arrows*, SBB); ultrastructure of the hypochile epidermal cells in the 1st day. **c**, **d** Strongly distended cuticle on outer tangential walls with residues of secreted substances (*arrows*) on its surface and in the subcuticular space. **d**, **e** The lipoid globules present in the outer tangential walls of epidermis and in subcuticular space, the secreted material (*arrows*). **f** The lipid bodies in cytoplasm. **g** A few amount of secreted material (*arrows*) on the cuticle surface, crossing the cuticle and as micro-channels, the irregular plasmalemma and periplasmic space (*white arrows*). **h** The cell walls (outer tangential, inner tangential, anticlinal) of epidermis with numerous globules, probably lipid bodies, abundant spherical or prolonged mitochondria. **i** Plastid with electron-dense body, intraplastid membrane system and starch grain. *c* cuticle, *cw* cell wall, *l* lipid drop, *m* mitochondrion, *p* plastid, *RER* rough endoplasmic reticulum, *ss* subcuticular space, *va* vacuole

**Fig. 7:**
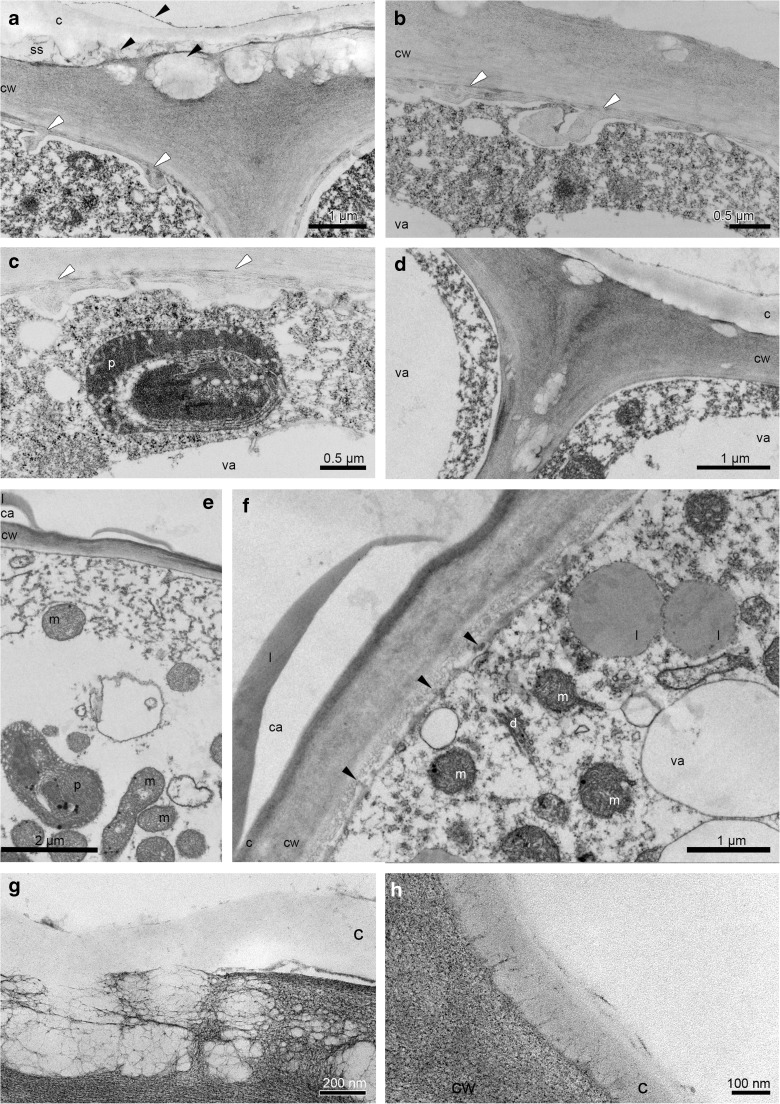
The pollinated flowers. Ultrastructure of the hypochile epidermal cells in the 3rd day of anthesis (**a**–**c**) and the 5th day (**d**). **a** The residues of secreted material on the cuticle, in the subcuticular space and in the cell wall (also **d**). **a**–**c** The developed periplasmic space (*white arrows*). **c** The oval-shaped plastids with intraplastid membrane system. Ultrastructure of the knobs epidermal cells of epichile, the 1st day of anthesis. **e**, **f** The lipoid-carbohydrate secretion. **f** Vesicles fusing with plasmalemma and appeared in periplasmic space (*arrows*). Ultrastructure of lips in the 14th day. **g** The secretory material on the hypochile. **h** Few material on knobs. *c* cuticle, *ca *carbohydrate material, *cw* cell wall, *d* dictyosome, *l* lipid drop or lipoid material, *m* mitochondrion, *p* plastid, *ss *subcuticular space, *va* vacuole

After the pollination, the flowers were quickly withered. The starch grains were utilised during the anthesis. The test for presence of dihydroxyphenols revealed very slight reaction of plastids (probably plastoglobuli) and thin layer on cuticle (Fig. 6a). Sudan III and Sudan Black B (SBB) confirmed one or few lipid bodies per cell and the lipoid material on the surface (Fig. 6b). The epidermal cells of hypochile (the first day of anthesis, first locality) revealed strongly distended cuticle on the outer tangential walls with residues of secreted substances upon the cuticle (Fig. 6c–e) and in subcuticular space (Fig. 6c–d, f). The cell walls (outer tangential, inner tangential, anticlinal) of epidermis contained numerous globules, probably lipid bodies (Fig. 6d, e), located also in cytoplasm (Fig. 6f). In the flowers of the more shaded second locality, a few amount of secreted material was located on the cuticle surface, crossing the cuticle and as micro-channels (Fig. 6g). Moreover, the irregular plasmalemma and periplasmic space were visible (Fig. 6g–i). The cell walls (outer tangential, inner tangential, anticlinal) of epidermis contained numerous lipid bodies (Fig. 6h). The plasmodesmata traversed the anticlinal walls (not shown). The cell volume was occupied by dense cytoplasm with few small vacuoles (Fig. 6h) or mostly by a thin layer of cytoplasm and a large vacuole (Fig. 6g–i). The vacuoles sometimes contained flocculent material (Fig. 6f, h). The oval-shaped or irregularly formed plastids were homogenous with osmiophilic plastoglobuli, intraplastid membrane system and electron dense body and lacked starch or possessed a single grain per plastid profile (Fig. 6h, i). In the close neighbourhood, numerous spherical or prolonged mitochondria with well-developed cristae, often in groups, were located (Fig. 6h). Free ribosomes (Fig. 6g, h) and rough endoplasmic reticulum (Fig. 6i) were common. The third day of anthesis (second locality) revealed the secreted material on the cuticle surface, in subcuticular space and in cell walls (Fig. 7a), but in less quantity than in the first day in the first locality (compare with Fig. 6c–e). The periplasmic space was more developed (Fig. 7a–c). The noticeable difference concerned the fact that the plastids did not contain starch grains (Fig. 7c). The fifth day of floral lifespan exposed secretory material on the cuticle, globules in cell walls and disintegrated cytoplasm (Fig. 7d).

The epidermal cells of knobs in the first day of anthesis (first locality) were also secretory, with the residues of secreted substances on surface (Fig. 7e, f), exuded by vesicles fusing with plasmalemma and appeared in periplasmic space, which characterises the granulocrine type of secretion (Fig. 7f). However, the nature of secretion was heterogenous: lipoid-carbohydrate (Fig. 7e, f). The cell volume was characterised by lipid bodies, numerous mitochondria with well-developed cristae, plastids with osmiophilic plastoglobuli, intraplastid membrane system and electron dense body and lacked starch, dictyosomes and adjacent vesicles (Fig. 7f). The secretory material on hypochile (Fig. 7g) and knobs (Fig. 7h) were found on the surface also in the 14th day of flowering, however, in less quantity.

## Discussion

The morphological, histochemical and ultrastructural analysis of flowers collected at different stages of anthesis, pollinated and unpollinated, provides strong evidence to conclude that the shell-shaped hypochile and the knobs of epichile form the nectary. The secretory material on the knobs was in less quantity; however, the neutral red reaction for the presence of osmophores was poor. In our opinion, the scent comes from the aromatic constituents of the nectar and the epichile tissue and the apices of all tepals (osmophores). The comparison between pollinated and unpollinated flowers revealed that the pollinated flowers quickly withered. On the other hand, the floral lifespan of unpollinated flowers even lasted up to the 16th day of anthesis and it was observed the appearance of the heterogeneous lipoid–carbohydrate–phenolic secretion in the end of anthesis, which could intensify the scent perception and enhance pollinators to these flowers.

The secretory cells in hypochile and knobs formed single-layered epidermis and several layers of underlying parenchyma built by small, isodiametric cells with thin walls and dense cytoplasm, relatively large nuclei, supplied by collateral vascular bundles, which reflect the nature of nectaries (Nepi 2007). Nectar can be gathered under the cuticle and released by the cuticle rupture under the growing pressure to the exterior (Durkee 1983; Curry et al. 1991), which was observed in *E. helleborine*, as in *E. palustris* (Kowalkowska et al. 2015a). During the floral lifespan, the residues of the secreted material were higher on the hypochile cells. The heterogeneous nature of the secreted material (lipoid-carbohydrate) and lipid globules in the cell walls, as well as in the cytoplasm, were also localised in both studied *Epipactis* species. The number of lipid bodies was diminished during anthesis (not visible in the 5th day in Sudan III, only visible in TEM), which suggest their utilisation. In the orchid secretory cells, lipid bodies are present in the plastid neighbourhood, which suggest their participation in the production of exuded substances and their transport by ER profiles (Davies et al. 2005; Stpiczyńska et al. 2005) or vesicles (Kowalkowska et al. 2015a, b, 2017). Apart from sugars (glucose, fructose, sucrose), the nectar could contain lipids, amino or organic acids, enzymes, antioxidants, vitamins, mineral ions and secondary metabolites (Baker and Baker 1975; Galetto et al. 1998; Lüttge and Schnepf 1976). Lipids were frequently recorded in the nectary cells of other orchids (Figueiredo and Pais 1992; Stpiczyńska 1997; Stpiczyńska and Matusiewicz 2001; Stpiczyńska et al. 2004; Paiva 2009; Kowalkowska et al. 2012, 2017), and they are also regarded as physical equivalents of volatiles (Swanson et al. 1980; Pridgeon and Stern 1983; Curry et al. 1988; Kowalkowska et al. 2012).

In nectaries of about 12.6% of angiosperms (Frei 1955), the collateral vascular bundles are frequently described, e.g. in *E. palustris* (Kowalkowska et al. 2015a), in *B. wendlandianum* (Kowalkowska et al. 2015b) and in *Epipogium aphyllum* (Święczkowska and Kowalkowska 2015). The presence of collateral vascular bundles defines the transport through apoplast. The nectar constituents, sugar and water, are transported via sieve tubes to nectariferous cells, noted in *E. helleborine* (Fahn 2000; Pacini et al. 2003; Vassilyev 2003; Barrera and Nobel 2004), and carbohydrates were deposited as starch in the nectariferous cells, e.g. in *H. imbricata* (Stpiczyńska et al. 2005), then the starch is hydrolyzed during secretion (e.g. Pais and Figueiredo 1994; Stpiczyńska 1997; Stpiczyńska et al. 2005; Kowalkowska et al. 2015a, b). The presence of starch grains is a common feature of nectaries and osmophores (Stern et al. 1987; Pacini and Nepi 2007; De Melo et al. 2010). Starch is utilised as a source of energy for production of nectar and scent (Vogel 1990; Pacini and Nepi 2007; Nepi 2007). The abundant starch grains were gradually reduced from bud stage. In sectional profile, the plastids had one starch grain in the nectary cells at the beginning of anthesis, alike in *H. imbricata* (Stpiczyńska et al. 2005). Some plastids had polymorphic shapes, as in *H. imbricata* (Stpiczyńska et al. 2005) and *E. palustris* (Kowalkowska et al. 2015a), which were known as related to a starch depletion. During the anthesis, the number of plastoglobuli within the plastids increased, as in *E. palustris* (Kowalkowska et al. 2015a). The lipoid–carbohydrate–phenolic materials have been demonstrated in the end of anthesis. The phenolic material was the same as in plastoglobuli in the fifth day of anthesis; however, such little amount was difficult to prove by histochemical test on dihydroxyphenols. The role of plastoglobuli in the synthesis of volatile components has been broadly discussed (Wiśniewska et al. 2018 and literature therein). Fragrance constituents are possibly produced in plastoglobuli, and then transported to the intraplastidal membranes, crossing the plastid envelope to the profiles of ER or migrating independently and finally reaching the plasmalemma and to the cell exterior. Fahn (1979) proposed a model of nectar secretion, when the pre-nectar is transported through the symplast of the secretory parenchyma, then it is loaded to ER or dictyosomes. The vesicles originated from ER or dictyosomes are fused with irregular plasmalemma, and the nectar is released to the exterior. The features such as irregular plasmalemma, the secretory vesicles that fuse with it, fully developed dictyosomes, numerous profiles of ER indicate vesicle-mediated process of secretion, which were frequently reported in orchid secretory cells (i.e. Kowalkowska et al. 2015a, b). The remarkable feature was the presence of periplasmic space in the third day of anthesis, which was not reported previously in *E. palustris* (Kowalkowska et al. 2015a). In orchid flowers, it has been described in *Bulbophyllum levanae* and *B. nymphopolitanum* (Wiśniewska et al. 2018) and *Oncidium trulliferum* (Stpiczyńska and Davies 2008). The occurrence of periplasmic spaces is possibly associated with merocrine secretion, when after the transport of exudation outside the cell, the cell continues its secretory activity. The substances could be transported by vesicles to the periplasmic space via granulocrine secretion and then to the external surface (Fahn 1979; Pacini and Nepi 2007; Paiva 2016). The transfer to the exterior may be facilitated by the apoplastic step—cuticular micro-channels of outer tangential (= periclinal) walls (Radice and Galati 2003). Cuticular canals (micro-channels) were reported in hypochile and knob cells. Both micro-channels and slightly developed periplasmic space were visible in the hypochile epidermis. Such co-existence was distinctly shown in *B. levanae* (Wiśniewska et al. 2018). However, in many nectaries, the way of pre-nectar transport could be simultaneous: both apoplastic and symplastic pathways (Davis et al. 1986, 1988; Kronestedt and Robards 1987; Razem and Davis 1999). The term ‘pre-nectar’ refers to the substances transported via plasmodesmata into the symplast of nectary tissue and then transformed into nectar in parenchyma nectary (Nepi 2007). The plasmodesmata in anticlinal walls were also visible in epidermis of *E. helleborine*, which is the character for transport via the symplast*.* On the other hand, the apoplastic route is continued as a continuum of cell walls. The carbohydrates transported to the nectary phloem may simultaneously be transferred to the nectary surface by a combination of these routes.

Mitochondria were abundant macroorganelle in floral nectaries in *E. helleborine*, which is associated with high metabolic cell activity, often existing in groups. Their number suggests the eccrine secretion, where the produced energy is used in active transport of pre-nectar carbohydrates to cross the plasmalemma (Eriksson 1977; Davis et al*.* 1986; Razem and Davis 1999).

The large amount of nectar secreted during anthesis, high temperatures during summer and alcohol present in scent profile could also suggest the occurrence of nectar fermentation. The alcoholic fermentation changes the sugar profile of nectar by reducing the total concentration of sugar and decreasing the amount of sucrose (Herrera et al. 2009; Canto et al. 2011). As the nectar composition is precisely related to pollinator preferences (Baker and Baker 1982; Raguso 2004), this change may influence the plant–pollinator relationship. Nevertheless, to the proposed hypothetical scheme of the chemical compounds’ influence on the pollinators and visitors insects of *E. helleborine* (Jakubska et al. 2005b), we would add the third stage: in the last day of anthesis in unpollinated flowers—discharge the nectar (especially on the knobs—Figs. 4h and 5a, b) and appearance of phenolic material on cuticle surface, which could attract pollinators by the intensification of the scent perception.

In all tepals, numerous idioblasts with raphides of calcium oxalate were visible, which possibly help to prevent herbivory (Prychid and Rudall 1999). Idioblasts located close to secretory tissue often occur in orchids (Wiśniewska et al. 2018 and literature therein).

We believe that the meticulous knowledge of floral architecture and production of attractants for pollinators will give more data that will be useful for future projects of plant conservation planning.
